# An RFID Tag Movement Trajectory Tracking Method Based on Multiple RF Characteristics for Electronic Vehicle Identification ITS Applications

**DOI:** 10.3390/s23157001

**Published:** 2023-08-07

**Authors:** Ruoyu Pan, Zhao Han, Tuo Liu, Honggang Wang, Jinyue Huang, Wenfeng Wang

**Affiliations:** 1 School of Communications and Information Engineering and School of Artificial Intelligence, Xi’an University of Posts and Telecommunications, Xi’an 710121, China; panruoyu@xupt.edu.cn (R.P.); hanzhao@stu.xupt.edu.cn (Z.H.); liutuo_99@stu.xupt.edu.cn (T.L.); wanghonggang@xupt.edu.cn (H.W.); 2 School of Science, Xi’an Shiyou University, Xi’an 710065, China; 202201060104@stumail.xsyu.edu.cn; 3 China Electronics Standardization Institute, Beijing 100007, China

**Keywords:** electronic vehicle identification, UHF RFID, multiple RF characteristics, trajectory tracking

## Abstract

Intelligent transportation systems (ITS) urgently need to realize vehicle identification, dynamic monitoring, and traffic flow monitoring under high-speed motion conditions. Vehicle tracking based on radio frequency identification (RFID) and electronic vehicle identification (EVI) can obtain continuous observation data for a long period of time, and the acquisition accuracy is relatively high, which is conducive to the discovery of rules. The data can provide key information for urban traffic decision-making research. In this paper, an RFID tag motion trajectory tracking method based on RF multiple features for ITS is proposed to analyze the movement trajectory of vehicles at important checkpoints. The method analyzes the accurate relationship between the RSSI, phase differences, and driving distances of the tag. It utilizes the information weight method to obtain the weights of multiple RF characteristics at different distances. Then, it calculates the center point of the common area where the vehicle may move under multi-antenna conditions, confirming the actual position of the vehicle. The experimental results show that the average positioning error of moving RFID tags based on dual-frequency signal phase differences and RSSI is less than 17 cm. This method can provide real-time, high-precision vehicle positioning and trajectory tracking solutions for ITS application scenarios such as parking guidance, unmanned vehicle route monitoring, and vehicle lane change detection.

## 1. Introduction

EVI is a technology used for vehicle identification and data exchange. It employs electronic tags installed on vehicles for wireless communication and information transmission. In ITS, vehicle trajectory tracking, and behavior recognition are crucial for improving traffic management efficiency, ensuring traffic safety, and optimizing vehicle dispatching.

RFID can provide a more accurate and reliable way to track and monitor the position of tags within specific areas. RFID tags can provide real-time location data of vehicles to be obtained and their travel trajectories to be recorded, thus providing valuable support for traffic planning and decision making.

The tag positioning method based on RSSI uses the free-space propagation model or path loss model as the principle, and the location of the tag to be measured is estimated by measuring the tag’s RF signal strength using a reader [1]. Classic algorithms based on RSSI include SpotON, LANDMARC, and VIRE [2,3,4]. Ref. [5] proposed a triangulation-based positioning method, while Zhao et al. [6] introduced a clustering-based positioning method. In [7], a probabilistic model is established for identifying transient key regions-OTrack. Yang et al. [8] proposed a method of locating the target tag using nonlinear support vector regression and particle swarm optimization. However, in actual RFID applications, RSSI is not a reliable positioning indicator due to environmental noise and multipath interference. Refs. [9,10] used the fingerprint library method to mitigate the fluctuations of RSSI signals caused by multipath effects and changes in labeling methods. Fingerprint datasets can be pre-collected as a historical database or built using reference tags together with the target tag. The position of the target tag is calculated based on the coordinates of multiple reference tags selected from the database using matching techniques. In [11], Wang et al. utilized phased array antennas to measure the azimuth angle of the target tag, read the signal strength, and utilized neural networks to fit the distance curve for tag positioning.

In recent years, the tag positioning method based on phase has gained increasing attention. The monotonic variation range of phase is half a wavelength, which is too short in UHF RFID systems and leads to phase ambiguity, making it unsuitable for direct use. Nikitin et al. [12] provided a comprehensive analysis of three main methods based on the phase difference of arrival (PDOA): time domain (TD), frequency domain (FD), and spatial domain (SD). Among them, FD-PDOA estimates the distance between the antenna and the tag by measuring the phase at different frequencies. In [13], Thangarajah et al. overcame the limitation of phase wrapping by measuring the round-trip flight time and unwrapping the phase. Ref. [14] calculated the radial velocity of RFID tags by measuring the phase difference between two consecutive time steps. Refs. [15,16] investigated the range estimation of RFID tags using multiple frequencies. In [17], a novel approach is proposed, which uses phase differences with multiple antennas to track the moving UHF RFID tags in a three-dimensional space. Wu et al. [18] established a phase unwrapping model and utilized a moving antenna to obtain the position of static tags. Ref. [19] achieved fine-grained positioning by using phase difference information measured by multiple antennas, making it possible to detect human activities at natural speeds by measuring the phase difference between pulses within the same pulse. In [20], Chatzistefanou et al. utilized the measured phase differences for each antenna, combined them with hyperbolic curve fitting to calculate possible tag positions, evaluated the distance between the trajectory and hyperbolic curves, and selected the most suitable trajectory for the moving tag.

In addition, constructing a spatial likelihood map can infer the initial position and the moving trajectory. In [21], RF holography is introduced to compute the spatial likelihood distribution, primarily used in the fields of ultrasound and radar imaging. Ref. [22] proposed Track-T to incorporate phase periodicity to extract the initial position of the trajectory. Another emerging wireless positioning method is RTI [23], which utilizes the shadow fading of wireless signals to construct attenuation images of objects within the monitoring area. Ref. [24] proposed a hybrid attention semantic segmentation network for the surveillance of area use, which can extract the target and its surroundings through a large receptive field for multi-scale targets.

The vehicle positioning schemes based on UHF RFID technology have attracted considerable attention [25,26,27]. In [28], Chen et al. utilized a single-antenna multi-frequency ranging scheme in UHF RFID, resolving phase ambiguity using the robust Chinese Remainder Theorem (CRT) based on maximum likelihood estimation, and achieving vehicle positioning without GPS. In [29], a road hazard detection solution based on a cooperative vehicle infrastructure system is proposed for road hazard detection. The method can effectively detect road hazards and obtain an accuracy of 90.2% with an inference time of 14.7 ms. To improve the performance of existing roadside unit (RSU) access schemes, Li et al. proposed a BUS-aided RSU connection scheme based on software-defined networking (SDN) and evolutionary game theory. This method improves the efficiency of the mac layer for RSU access [30].

In [31], Tzitzis et al. achieved real-time localization of mobile robots by combining sensors and RFID technology. However, this method did not optimize the positioning error of RFID technology itself. Instead, it used a fingerprinting technique to improve position accuracy by comparing the returned values of reference tags with position information and external sensor data. The utilization of tag characteristics was not fully exploited in this method. In [32], Liu et al. designed a novel reader and used the virtual frequency difference to optimize the PDOA positioning error, achieving high-precision localization. This method calculates the distance differences based on phase differences and introduces hyperbolic positioning. However, the application scope of this method is relatively limited, and it did not consider the impact of time delay between measured phase differences when the vehicle is moving at high speeds and the distance the vehicle covers. Additionally, if the virtual frequency difference is too significant, the reading distance of the reader will significantly decrease to ensure the phase difference remains within the 2π range. This paper proposes a trajectory tracking method based on multiple characteristics of UHF passive RFID electronic tags. It focuses on the principles, positioning algorithms, system design, and experimental evaluation of RFID vehicle electronic tags. The method utilizes multiple RF characteristics to obtain more positioning information. This method significantly improves positioning accuracy and precision compared to a single RF characteristic position. Furthermore, it enhances the system’s robustness and reliability, enabling effective operation under different environmental conditions and mitigating positioning errors caused by signal attenuation, multipath interference, and other factors. By analyzing the trajectory of electronic identifications, this method provides essential insights into the vehicle driving behavior and trajectory patterns for intelligent transportation systems.

The remaining sections of this paper are organized as follows. In Section 2, an analysis of the parameter characteristics of the moving tags is conducted, including the investigation of their variations with changes in the antenna–tag distance (the distance between the reader antenna and the tag, referred to as the antenna–tag distance). Additionally, filtering techniques are applied to the tag parameters. In Section 3, a detailed explanation of the tracking method is provided. This method utilizes a single antenna for distance estimation and multiple antennas for positioning. The coordinates of the positioning area are calculated to determine the location of the tag. Section 4 presents experimental evaluations of the proposed method, verifying the tracking method in scenarios involving both linear and curved movements of the tags. Finally, the conclusions are summarized in Section 5.

## 2. Characteristics of Tag Parameter Information

### 2.1. Preprocessing of RSSI

#### 2.1.1. Multipath Effect of RSSI

**Figure 1:**
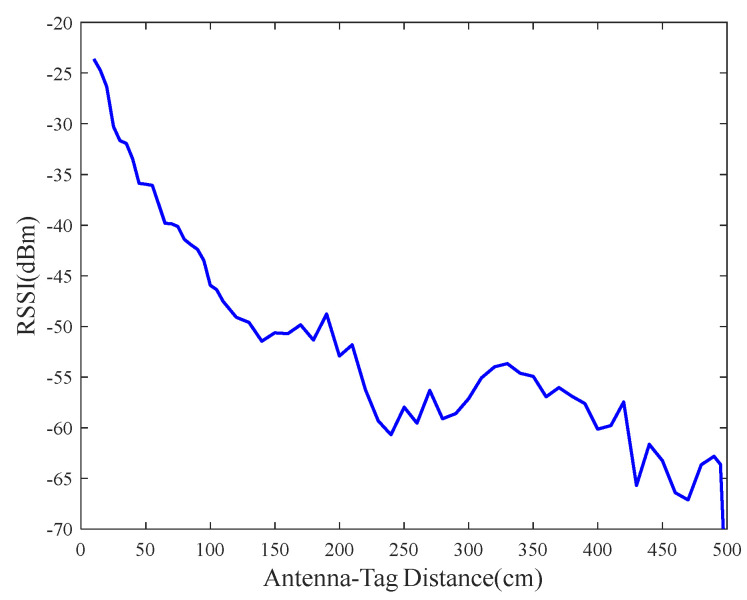
The tag return power with antenna–tag distance at a reader transmit power of 30 dBm.

Due to the complexity of the environment, there are numerous refractions, scattering, and diffraction, which lead to the influence of multipath effects on the RSSI. As a result, there is no one-to-one mapping between the RSSI and antenna–tag distance, which is shown in Figure 1. The RSSI corresponds to multiple distances at longer distances, and the monotonicity is lost. To establish a unique correspondence between RSSI and the antenna–tag distance, the preprocessing of the RSSI measurements is required.

#### 2.1.2. Distribution Properties of RSSI

**Figure 2:**
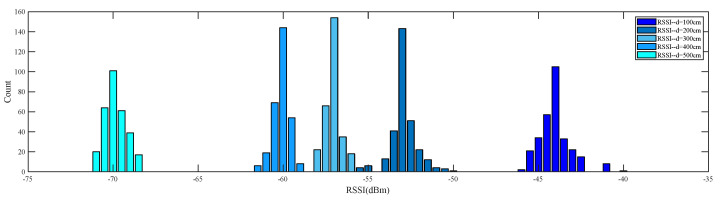
Characteristics of RSSI intensity distribution at different antenna–tag distances.

The RSSI collected from the same tag by the same reader over continuous time may contain invalid data due to the multipath effects and noise interference. The distribution characteristics of the measured RSSI at fixed points follow a Gaussian distribution [14,33]. Figure 2 shows the distribution of measured RSSI at distances of 100 cm, 200 cm, 300 cm, 400 cm, and 500 cm from the antenna. Here, RSSI is preprocessed by Gaussian filtering. The Gaussian model selects the RSSI of the interval of a high probability of occurrence as the valid value. Then, its average value is calculated as the filtered data for positioning to reduce the influence of the environment on the accuracy of RSSI.

The noise of the measured RSSI obeys the Gaussian distribution of $\left(0,\sigma^{2}\right)$, and its probability density function (PDF) is given by

(1) $$f_{RSSI}=\frac{1}{\sigma \sqrt{2\pi }}\cdot e^{\frac{{\left(RSSI-\mu \right)}^{2}}{2\sigma^{2}}}$$

where $\mu =\frac{1}{n}\cdot \sum_{k=1}^{n}RSSI_{k},\sigma =\sqrt{\frac{1}{n-1}\cdot {\sum_{k=1}^{n}\left(RSSI_{k}-\mu \right)}^{2}}$

(2) $$\bar{RSSI}=\frac{1}{N}\cdot \sum_{k=1}^{N}RSSI_{k},RSSI_{k}\in (\mu -k\sigma ,\mu +k\sigma )$$

The mathematical model of the relationship between $\bar{RSSI}$ and antenna–tag distance is obtained by Gaussian filtering and calculating the fixed point.

### 2.2. Preprocessing of Phase

**Figure 3:**
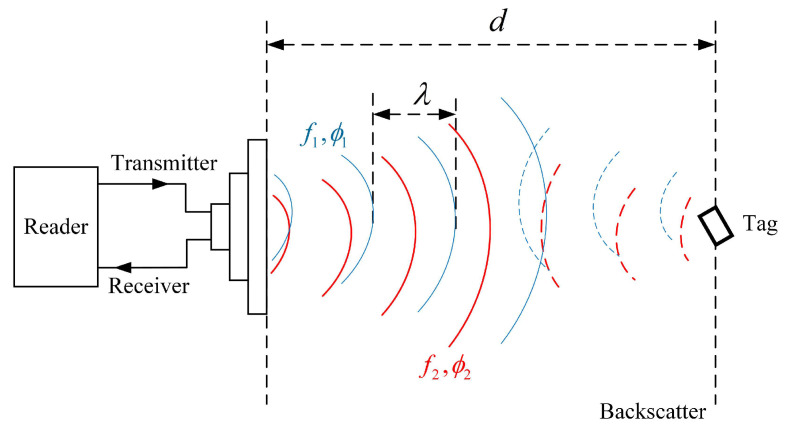
Diagram of the backscatter radio link of the RFID communication.

Data communication between UHF passive RFID tags and readers is achieved using a backscatter radio link. As shown in Figure 3, d is the antenna–tag distance and λ is the wavelength; the RFID antenna controlled by the RFID reader actively transmits RF signal to the tag. The tag receives commands and requires power from the RF signal. Once the communication link is established, the tag modulates its data by changing its load impedance and reflects the modulated RF signal to respond to the commands of the reader.

#### 2.2.1. Phase Ambiguity

The measured phase of the backscattered signal of the tag is wrapped in [0,2π]. In addition, the measured phase always includes the superposition of the noise and the phase returned from the surrounding scattering. The mathematical expression for the measured phase is given by

(3) $$\{\begin{array}{c} \phi_{m}=(\phi_{p}+\phi_{o})mod(2\pi )\\ \phi_{p}=\frac{4\pi }{\lambda }d\\ \phi_{o}=\phi_{T}+\phi_{R}+\phi_{Tag} \end{array}$$

where $\phi_{m}$ is the measured phase, $\phi_{p}$ is the cumulative phase of the signal propagation, and $\phi_{o}$ is the constant phase shift generated by the reader transmitter circuit, receiver circuit, and tag, which is a distance-independent term. λ is the RF signal wavelength, and d is the antenna–tag distance.

**Figure 4:**
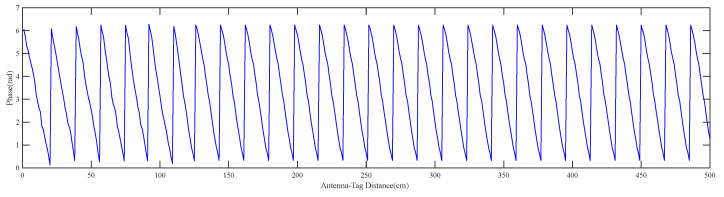
Variation characteristics of phase with antenna–tag distance.

Equation (3) shows that the measured phase $\phi_{p}$ is the cumulative phase shift of the backscattered signal modulo 2π, which means the phase of the received signal has a periodicity of 2π. To provide a more intuitive representation, consider a scenario where a tag moves away from the antenna in a straight line at a constant velocity. The phase variation in the tag can be visualized as shown in Figure 4.

Figure 4 shows that with the variation in the antenna–tag distance, the measured phase exhibits stronger stability at longer distances compared to RSSI. The phase of the received signal is wrapped and repeated within [0,2π]. Therefore, the measured phase alone cannot directly reflect the spatial antenna–tag distance, which is called phase ambiguity. In Section 2.2.3, the FD-PDOA is employed to eliminate the periodic phase ambiguity of 2π, establishing a unique correspondence between the measured phase and the antenna–tag distance.

#### 2.2.2. Distribution Properties of Phase

**Figure 5:**
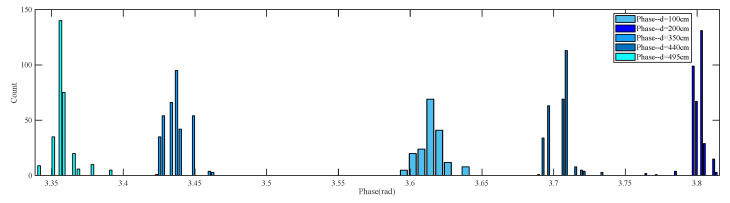
Measured phase distribution at different antenna–tag distances.

The measured phase always contains random errors caused by reader thermal noise and multipath effects [15,16,18]. Figure 5 depicts the distribution of measured phase for antenna–tag distances of 100 cm, 200 cm, 350 cm, 440 cm, and 495 cm, with the RFID reader operating at a frequency of 925.75 MHz. It can be observed that the measured phase also follows a Gaussian distribution.

Similarly, the noise of the measured phase obeys the Gaussian distribution of $\left(0,\sigma^{2}\right)$, and its PDF is given by

(4) $$f(\phi_{m})=\frac{1}{\sigma \sqrt{2\pi }}\cdot e^{\frac{{\left(\phi_{m}-\mu \right)}^{2}}{2\sigma^{2}}}$$

where $\mu =\frac{1}{n}\cdot \sum_{k=1}^{n}\phi_{mk},\sigma =\sqrt{\frac{1}{n-1}\cdot \sum_{k=1}^{n}{\left(\phi_{mk}-\mu \right)}^{2}}$.

#### 2.2.3. FD-PDOA

**Figure 6:**
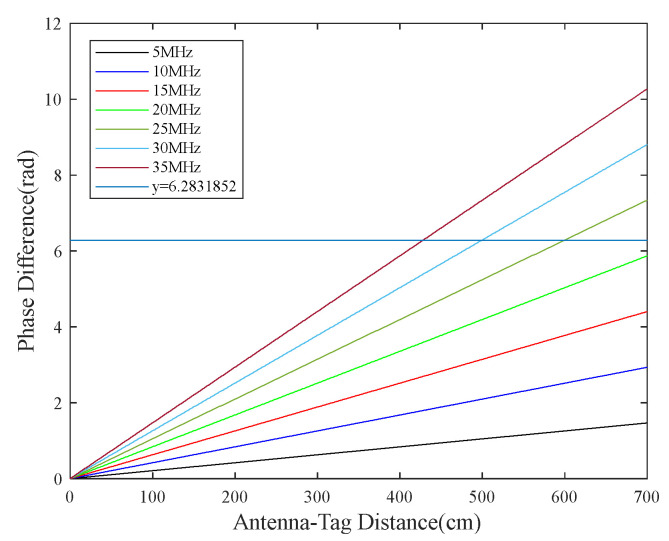
Relationship between phase difference and distance at different frequency differences.

FD-PDOA estimates the antenna–tag distance by measuring the tag phase under different frequencies, and its principle is shown in Figure 3, and the mathematical expression is given by

(5) $$d=\frac{c\cdot \Delta \phi }{4\pi \cdot \Delta f}$$

where $\Delta \phi =\phi_{2}-\phi_{1}$, $\Delta f=f_{2}-f_{1}$, and c represents the speed of electromagnetic wave propagation. The noise in Δϕ follows $N(0,\sigma_{1}^{2}+\sigma_{2}^{2})$. Given the determined frequency difference in Δf, the phase difference is directly proportional to the distance d. The variation in the phase difference with distance is shown in Figure 6. Different values of Δf result in different magnitudes of phase difference at the same distance. Moreover, the phase difference can exceed 2π as the distance increases, indicating the presence of phase ambiguity. Therefore, it guarantees the antenna identification area meets the actual demand ($d_{m}$) and guarantees Δϕ≤2π, $\Delta f=\frac{c\cdot 2\pi }{4\pi \cdot d_{m}}$, where Δf belongs to the frequency hopping interval provided by the reader.

The reader has K frequency points in its operating band, corresponding to K(K−1)/2 phase differences.

(6) $$d^{i}=\frac{c\cdot \Delta \phi_{i}}{4\pi \cdot \Delta f_{i}},i=1,2,......,K(K-1)/2$$

(7) $$\bar{d}=\frac{\sum_{i=1}^{K(K-1)/2}d_{i}}{K(K-1)/2}$$

In Equation (6), $d^{i}$ is the distance corresponding to the i th phase difference, and in Equation (7), $\bar{d}$ is the average of the distances corresponding to K(K−1)/2 phase differences, and $\bar{d}$ converges to the actual antenna–tag distance d.

## 3. Mobile Tag Trajectory Tracking Method

The RSSI has good monotonicity at close range, and the phase difference has good anti-interference at long range. Using the characteristics of RSSI and phase difference to achieve tag estimation distance, the tag is clustered according to the estimation distance accuracy of the tag parametric information, and the weight distribution is used to optimize the distance more accurately. The possible position of the tag is located on the circle with the antenna as the center and the estimated range as the radius, and the actual result is a circle with the estimated range plus or minus the estimated range error as the inner and outer radius. In this paper, the position overlap area of four reader antennas in different directions is used to calculate the tag position.

### 3.1. UHF RFID Vehicle Trajectory Tracking Model

**Figure 7:**
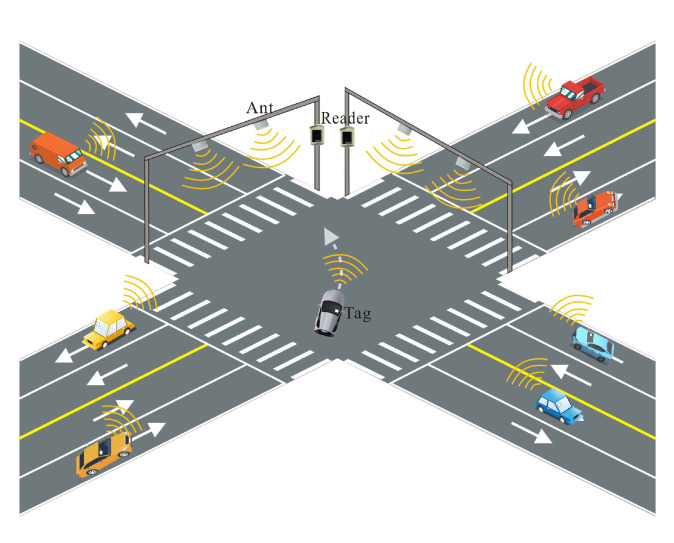
UHF RFID vehicle trajectory tracking model.

As shown in Figure 7, the RFID system can be used as a roadside checkpoint in the vehicle trajectory tracking method proposed in this paper. These checkpoints consist of RFID antennas and readers installed on road facilities, which automatically read and identify the electronic tags of passing vehicles within the antenna coverage area. This enables the tracking and monitoring of vehicle trajectories. In addition to roadside checkpoints, the proposed method is also applicable to other scenarios. For example, unmanned vehicles in a multipath operational area can carry RFID tags and can be tracked using the RFID system for real-time position monitoring and path planning. Furthermore, the RFID system can be used as checkpoints to track and manage vehicle trajectories in enclosed environments, such as factories, warehouses, and underground parking lots. This paper proposes a general mobile tag trajectory tracking method suitable for various application scenarios, providing a unified solution for vehicle trajectory tracking in different fields.

### 3.2. Tag Parameter Estimation Distance Procedure

#### 3.2.1. RSSI Estimation Distance Procedure

By obtaining the variation pattern of RSSI with antenna–tag distance, the mathematical model of signal strength versus distance is given by

(8) $$d_{RSSI}=\{\begin{array}{c} d_{RSSI}^{1}=f(RSSI_{1})\\ d_{RSSI}^{2}=f(RSSI_{2})\\ \vdots \\ d_{RSSI}^{i}=f(RSSI_{i}) \end{array}$$

where $d_{RSSI}^{i}$ is the estimated distance from the antenna i to the mobile tag.

#### 3.2.2. Phase Difference Estimation Distance Procedure

**Figure 8:**
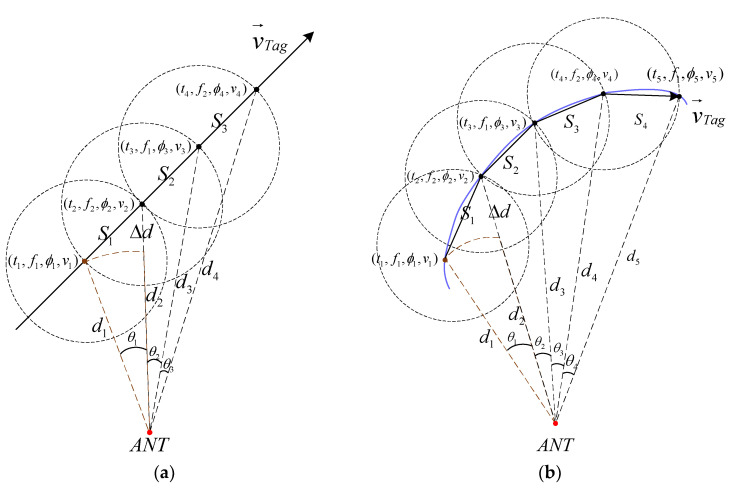
Distance incremental compensation chart: (**a**) is a tag that makes a uniform linear motion; (**b**) is a tag that makes a uniform curve motion.

Equation (5) assumes a unique value for d. However, for a moving tag, there is a time delay, $\Delta t=t_{2}-t_{1}$, and a distance traveled $S=\int_{t_{1}}^{t_{2}}vdv$ when obtaining the tag information at Δf. To satisfy the requirements of the FD-PDOA, an incremental compensation for the distance, denoted as $\Delta d=d_{2}-d_{1}$, needs to be added to Equation (5). This is shown in Figure 8a, where the tag is moving in a straight line. The red point represents the position of the antenna, the brown point represents the initial position of the tag, and the solid black line represents the trajectory of the tag’s movement, with the arrow indicating the direction of motion. Assuming that the initial position of the tag at $t_{1}$ is known, and at $t_{2}$, the tag is located on a circle with the initial position as the center and a radius of S, and the angle between the tag and the antenna at $t_{1}$ and $t_{2}$ is denoted as θ. Therefore, the cosine expression for $d_{2}$ at $t_{2}$ is given by

(9) $$S^{2}=d_{1}^{2}+d_{2}^{2}-2d_{1}d_{2}cos\theta$$

(10) $$d_{2}=\sqrt{S^{2}-d_{1}^{2}sin^{2}\theta }+d_{1}cos\theta$$

Then,

(11) $$\Delta d=d_{2}-d_{1}=\sqrt{S^{2}-d_{1}^{2}sin^{2}\theta }+d_{1}cos\theta -d_{1}$$

The initial point $d_{1}$ can be easily obtained for fixed point ranging. Similarly, in Figure 8b, a tag moving along a curved trajectory can be divided into segments and calculated as straight lines. The distance traveled between the two time points is approximately equal to the straight-line distance S. The calculated distance d in the following equations refers to the distance at the second time point after obtaining the information of the tag parameter at two consecutive time points.

Equation (5) after the distance increment is given by

(12) $$d=f(t,f,\phi ,v)=\frac{c\cdot [\phi_{t_{2}}^{1}-(\phi_{t_{1}}^{1}+\frac{4\pi f_{1}\cdot \Delta d}{c}mod(2\pi ))]}{4\pi \cdot \Delta f}$$

where $\phi_{t_{1}}^{1}$ represents the phase measurement of antenna 1 at time $t_{1}$ and $f_{1}$, and $\phi_{t_{2}}^{1}$ represents antenna 1 at time $t_{2}$ and $f_{2}$. By calculating the phase change under the distance of the absolute location increment Δd of the tag, let it take the remainder for 2π, add $\phi_{t_{1}}^{1}$ to the result of the remainder to obtain the corresponding phase at times $t_{2}$ and $f_{1}$, which satisfies the condition of Equation (5).

In Equation (13), $d_{Phase}^{i}$ is the estimated distance from the antenna i to the mobile tag.

(13) $$d_{Phase}=\{\begin{array}{c} d_{Phase}^{1}=f(t_{1},f_{1},\phi_{1},v_{1})\\ d_{Phase}^{2}=f(t_{2},f_{2},\phi_{2},v_{2})\\ \vdots \\ d_{Phase}^{i}=f(t_{i},f_{i},\phi_{i},v_{i}) \end{array}$$

### 3.3. Distribution of Weight Ratios

#### 3.3.1. Tag Parametric Estimation Distance Clustering

**Figure 9:**
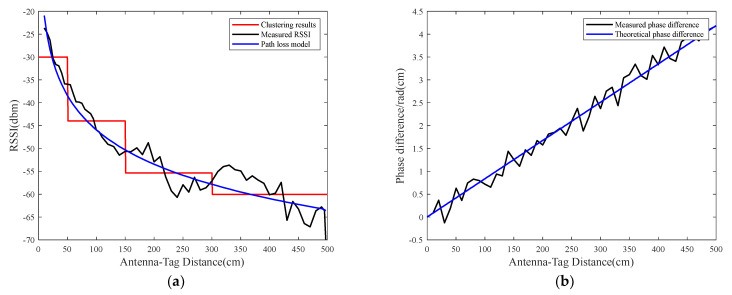
Measured and theoretical values of tag parameters: (**a**) represents a comparison of RSSI measured value and theoretical value; (**b**) represents phase difference variation at 20 MHz frequency difference. The classification result of the measured phase follows the measured RSSI.

In Section 3.2, we showed the obtained estimated distances of the moving tag based on the RSSI and phase difference. This section analyzes the accuracy of distance estimation using these two measured parameters. The K-MEANS clustering algorithm is utilized to cluster the comparison results between the estimation and true distances. Clustering is based on the corresponding accuracy of the distance estimation using these two measured parameters. The theoretical value of the RSSI estimation distance is obtained using the general formula of the spatial path loss model. The root mean square error (RMSE) between the RSSI distance estimate and the theoretical distance value corresponding to the RSSI in the spatial path loss model is calculated. Figure 9a shows the relationship between the measured RSSI and distance as a black line, the relationship between the theoretical RSSI and distance obtained from the path loss model as a blue line, and the clustering result as a red line. The general formula for the spatial path loss model [14] is given by

(14) $$P=P_{0}-10n\cdot log(\frac{d}{d_{0}})$$

where P represents RSSI, d represents the distance between the node sending the signal and the node receiving the signal. $P_{0}$ is a reference value indicating the RSSI of the signal received by the receiving node from a node at a fixed distance $d_{0}$ from it, and n is the path loss index.

#### 3.3.2. Weighting of Parameter Estimation Distance Values

Section 2 states that the measured RSSI and phase difference at any fixed point follows Gaussian distributions. Specifically, the RSSI at a fixed point follows a Gaussian distribution with parameters $\left(0,\sigma^{2}\right)$, and the phase difference follows a Gaussian distribution with parameters $\left(0,\sigma_{1}^{2}+\sigma_{2}^{2}\right)$. Furthermore, the variations in RSSI and phase difference with distance are modeled as Gaussian processes. To evaluate the ranging accuracy of the parameter information, the RMSE between the estimated distance values and the actual distances is calculated. The information weighting method is used to assign weights, and the weighted estimated distance is given by

(15) $$d^{i}=\omega_{RSSI}\cdot d_{RSSI}^{i}+\omega_{Phase}\cdot d_{Phase}^{i}$$

where $\omega_{i}=\frac{CV_{i}}{\sum CV_{i}}$, CV is the coefficient of variation.

### 3.4. Position Calculation of Tag

**Figure 10:**
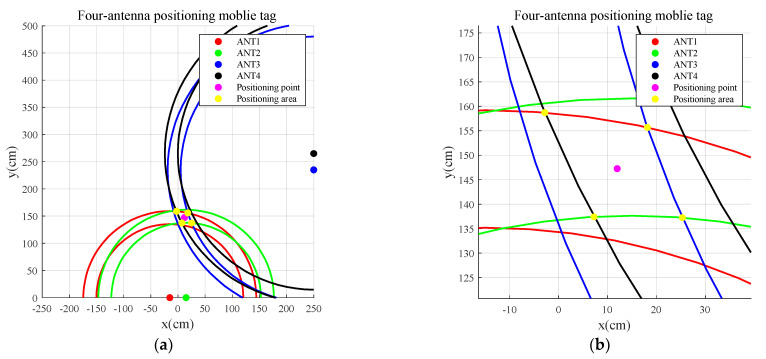
Position area of the mobile tag: (**b**) is the enlarged view of (**a**).

In Section 3.3.2, we showed the weighted estimated distance value, $d^{i}$. The tag is located in the region of $d^{i}\pm d_{error}$, which means the tag is within a circular ring with the antenna center as the center, an inner radius of $r_{i}=d^{i}-d_{error}$, and an outer radius of $R_{i}=d^{i}+d_{error}$. This corresponds to the common region of n antennas and n circular rings. In this section, the positions of the antennas and the tag’s movement area are visualized using a coordinate system while avoiding overall offset in the test results, as shown in Figure 10.

The Cartesian coordinate system is defined as follows: the *x*-axis passes through the line connecting two antennas, and the *y*-axis passes through the midpoint of the two antennas.A,B,C,D represent the positions of the four antennas, with coordinates $\left(x_{0},y_{0}\right)$, $\left(-x_{0},y_{0}\right)$, $\left(x_{1},y_{1}\right)$, and $\left(x_{1},y_{2}\right)$, respectively. The tag T moves within the effective range of the antennas. At any given time t, the tag is located within the common region of the four circular rings, as shown in Figure 10. The common region of the four circular rings forms a quadrilateral with yellow points as vertices. The purple point at the midpoint of the common region represents the position of the tag. The calculation process is as follows.

By introducing the weighted estimated distance value, $d^{i}$, into the coordinate system in Equation (15), the expression for the circle $CC_{i}$ is given by

(16) $$CC_{i}=\{\begin{array}{c} {\left(x-x_{i}\right)}^{2}+{\left(y-y_{i}\right)}^{2}=r_{i}^{2}\\ {\left(x-x_{i}\right)}^{2}+{\left(y-y_{i}\right)}^{2}=R_{i}^{2} \end{array},i=1,2,......,n$$

To calculate the common region of the rings, we perform the intersection calculation two by two for the circles in the n rings; concentric circles do not need to compute the intersection points. The execution results in a matrix of size n×n $C_{ij}$ denotes the intersection of two circles, $C_{ij}=C_{ji}$, and the set of all intersection points of the circles intersecting two by two is given by

(17) P{Cij(xi,yi)}={(x0,y0),(x1,y1),......(xN,yN)|i=0,1,2,......,N}

Intersection filter condition:

1. $y>y_{0},-x_{1}<x<x_{1}$.
2. The distance from any point in the common area of the four circles to the center of any circle is within the inner and outer radius of the circle.

The set of intersection points filtered by conditions 1 and 2 is given by

(18) P{Cij(xi,yi)}Aera={(x0,y0),(x1,y1),......(xM,yM)|M≤N}

The elements of the set P{Cij(xi,yi)}Aera are the vertices of the common region of the n circles, and the M intersection points represent the convex edge of the common region with M sides. The coordinates of the midpoint of the convex polygon is calculated by using the following formula:

(19) {X=(∑i=0Mxi)/(M+1)Y=(∑i=0Myi)/(M+1)

Then, the calculation process of tag position confirmation is summarized in Algorithm 1.
**Algorithm 1:** Mobile Tag Positioning Algorithm**Input:** $d^{i}$, $x_{i}$, $y_{i}$, $d_{error}$, i=1,2,...,n **Output:** X,Y$r_{i}=d^{i}-d_{error},R_{i}=d^{i}+d_{error}$${\left(x-x_{i}\right)}^{2}+{\left(y-y_{i}\right)}^{2}=r_{i}^{2}$${\left(x-x_{i}\right)}^{2}+{\left(y-y_{i}\right)}^{2}=R_{i}^{2}$**Calculate** $C_{ij},|i-j|\geq 1,r_{i},r_{j}\in \{r_{i},R_{i}\}$$d_{ij}=\sqrt{{\left(x_{i}-x_{j}\right)}^{2}+{\left(y_{i}-y_{j}\right)}^{2}}$If $|r_{i}-r_{j}|\leq d_{ij}\leq r_{i}+r_{j}${       $cosA=(r_{i}^{2}+d_{ij}^{2}-r_{j}^{2})/(2r_{i}\cdot d_{ij})$,$sinA=\sqrt{1-cos^{2}A}$       
$dx=(x_{i}-x_{i})/d_{ij}$
       
$dy=(y_{j}-y_{i})/d_{ij}$
       
x1=xi+ri⋅cosA⋅dx−ri⋅sinA⋅dy
       
y1=yi+ri⋅cosA⋅dy+ri⋅sinA⋅dx
       
x2=xi+ri⋅cosA⋅dx+ri⋅sinA⋅dy
       
y2=yi+ri⋅cosA⋅dy−ri⋅sinA⋅dx
   }P{Cij(xi,yi)}={(x0,y0),(x1,y1),......(xN,yN)|i=0,1,2,......,N}**If** −x1≤Cij(xi)≤x1 && $C_{ij}{(}_{i}y)\geq y_{0}${      **for; i<=len(***P***); i++** {            
D=(Cij(xi)−xi)2+(Cij(yi)−yi)2
                          **if** $r_{i}\leq D\leq R_{i}$ {                                  P{Cij(xi,yi)}Aera={(x0,y0),(x1,y1),......(xM,yM)|M≤N}
                            }               }     }X=(∑i=0Mxi)/(M+1), Y=(∑i=0Myi)/(M+1)**Return** X,Y

In the actual scene, the choice of coordinate system can be arbitrary when it is only necessary to translate and rotate the original coordinate system. If the original coordinate system after the rotation angle θ and translation are (a,b), and the coordinates of any point $T(x_{1},y_{1})$ of the original coordinate system in the target coordinate system are $T(x_{1}^{\cdot },y_{1}^{\cdot })$, then,

(20) $$\left[\begin{array}{c} x^{\cdot }\\ y^{\cdot } \end{array}\right]=\left[\begin{array}{cc} cos\theta & sin\theta \\ -sin\theta & cos\theta \end{array}\right]\left[\begin{array}{c} x_{1}\\ y_{1} \end{array}\right]+\left[\begin{array}{c} a\\ b \end{array}\right]$$

## 4. Experiments and Results

For the mobile tag tracking method studied in Section 3, this section uses the Impinj R420 reader with multiple circularly polarized antennas of size 25 × 25 cm with a gain of 9 dBi, along with the ISO 18000-6C tag, to verify its effectiveness.

### 4.1. Experimental Setups

**Figure 11:**
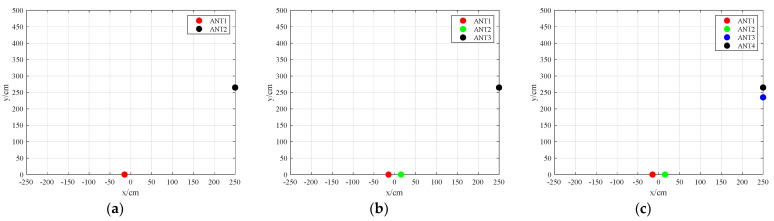
Antenna placement. (**a**) represents the placement of two antennas; (**b**) represents the placement of three antennas; and (**c**) represents the placement of four antennas. Specific coordinate is shown in Table 1.

The position of the antennas directly influences the effective identification area and position accuracy. In the experiment, the impact of antenna positions and quantities on the effective identification range and localization accuracy was evaluated, considering the overall system’s feasibility and cost. In this section, three antenna placement schemes are selected, as shown in Figure 11, where all three antennas are placed vertically. The advantages of this arrangement are two-fold: first, it minimizes the reduction in the common radiation area among multiple antennas. Second, the antennas placed along the *x*-axis constrain the *y*-axis range of the target tag, whereas the antennas placed along the *y*-axis constrain the *x*-axis range of the target tag. The detailed information regarding the antenna placement is provided in Table 1.

**Figure 12:**
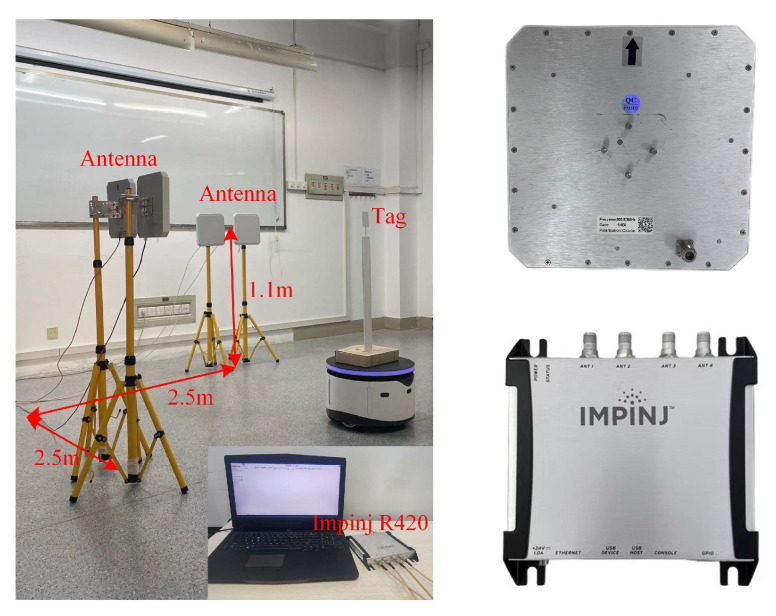
Experimental setup.

The experimental setup is shown in Figure 12. It consists of a fixed antenna within a 5 × 5 m indoor area, while the target tag is mounted on a stationary robot. The robot performs uniform linear motion, curved motion, and turnaround motion within the antenna’s identification range. The experimental parameters are listed in Table 1.

The placement with two antennas is shown in Figure 11a, the placement with three antennas is shown in Figure 11b, and the placement with four antennas is depicted in Figure 11c.

**Figure 13:**
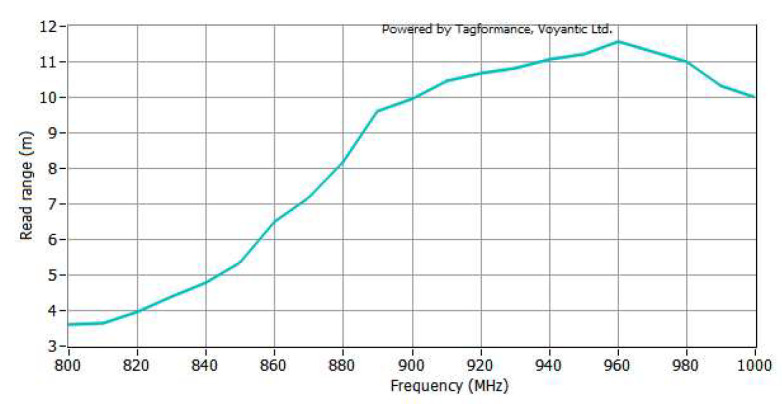
Read ranges of the tag: the read range is measured using the Voyantic Tagformance Pro comprehensive tester.

Figure 13 shows the read range of the tags used in this experiment. Within the frequency range of 902.75 MHz to 927.25 MHz, the read range of the tag can reach approximately 10.5 m.

### 4.2. Experimental Results

The experimental results show that the noise variance in the phase is about $\sigma_{i}^{2}\approx 0.1\;rad$. In the range of 0~500 cm, the estimation accuracy of the tag parametric information is classified into seven categories using the K-MEANS clustering algorithm, as shown in Table 2. In Equation (13), n=2.5, $d_{0}=100\mathrm{cm}$, and the standard signal strength value at $d_{0}$ is −45.76 dBm.

Table 3 shows the average RMSE obtained for each setup in all the experiments. The position accuracy using multiple characteristics is consistently higher than that of the single feature value localization results. Additionally, increasing the number of antennas improved localization accuracy based on multiple characteristics. When using four antennas to track the trajectory of moving tags with multiple feature values, the average RMSE in the experiments is minimized, measuring approximately 0.15 m.

The results of tracking the trajectory of the mobile tag using four antennas are as follows:

1. The robot with a tag makes a uniform linear motion.

**Figure 14:**
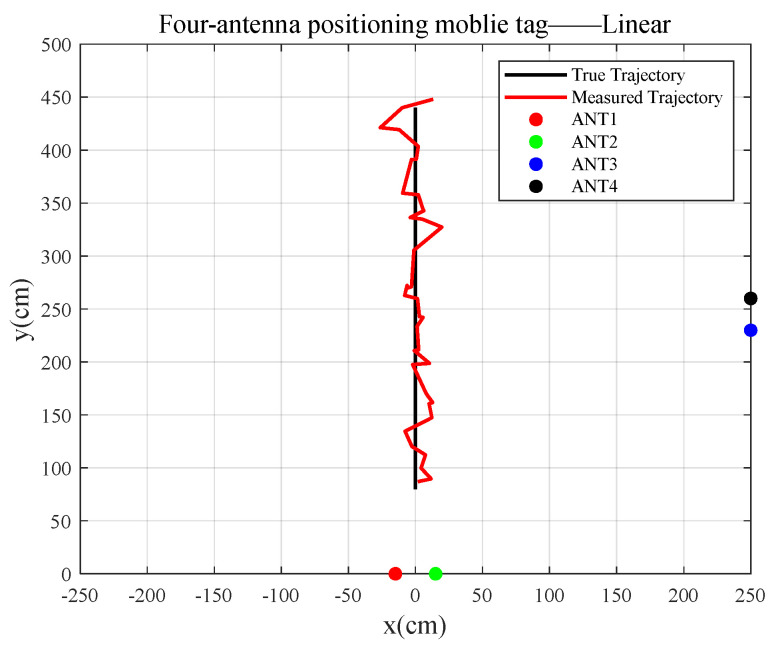
Tag uniform linear motion.

In Figure 14, the true moving trajectory of the uniform linearly mobile tag coincides with the calculated fitted trajectory, and the RMSE between the true trajectory and the fitted trajectory is 16.5262 cm.

2. The robot with a tag makes a uniform curve motion.

**Figure 15:**
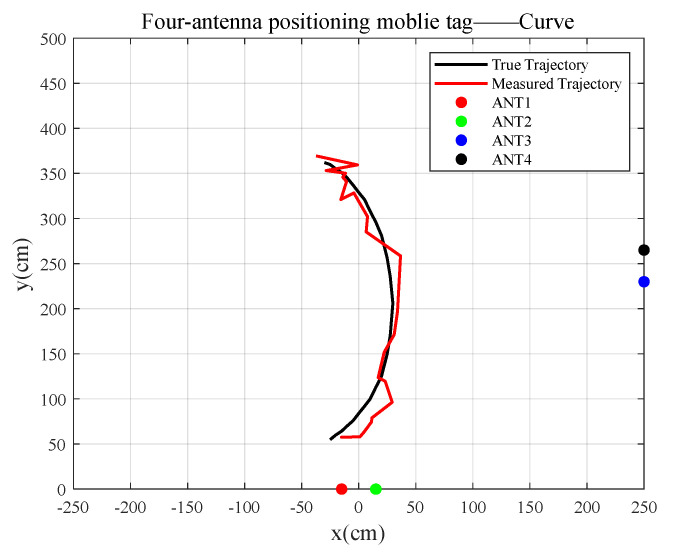
Tag uniform curve motion.

As shown in Figure 15, the true moving trajectory of the uniform curve mobile tag coincides with the calculated fitted trajectory, and the RMSE between the true trajectory and the fitted trajectory is 15.1935 cm.

3. The robot with a tag makes a uniform turnaround motion.

**Figure 16:**
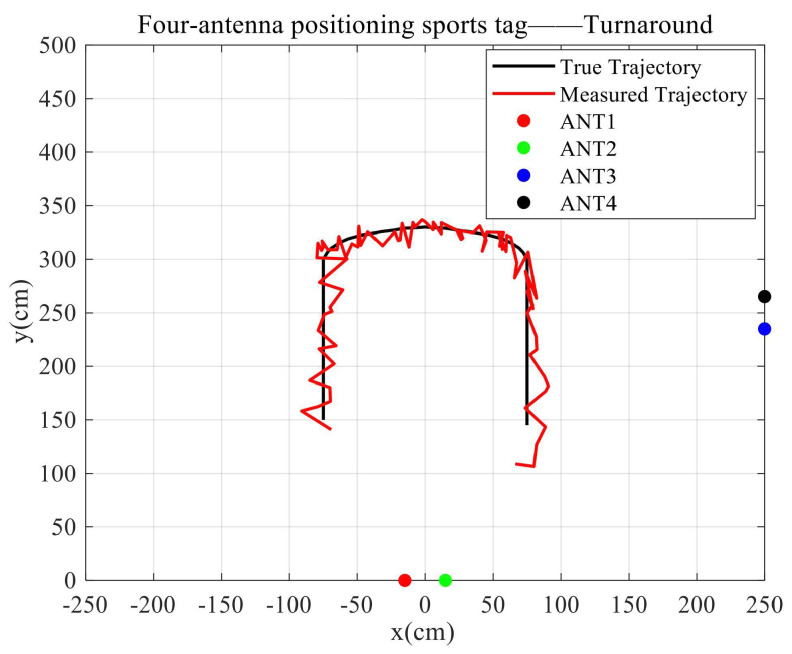
Tag uniform turnaround motion.

As shown in Figure 16, the true moving trajectory of the uniform turnaround mobile tag coincides with the calculated fitted trajectory, and the RMSE between the true trajectory and the fitted trajectory is 15.1972 cm.

## 5. Conclusions

This paper proposes a trajectory tracking method for UHF RFID vehicle electronic tags based on multiple RF features for ITS application scenarios. This method categorizes and assigns weights to multiple RF features by comparing the estimation accuracy obtained from a single feature value. The RSSI value obtained by multi-antenna measurement and the phase difference under dual-frequency conditions were used to determine the vehicle trajectory under the multi-antenna and multi-feature value scenarios. This method enables the real-time positioning of vehicle electronic tag tracking and backtracking of vehicle trajectory, analysis of vehicle driving status, and accurate, low-cost management of vehicles in specific areas. This paper utilizes traffic data collected via RFID to calculate vehicle trajectories, enabling real-time traffic flow monitoring, traffic information mining, and big data processing. It provides significant data support for predicting traffic congestion and formulating traffic plans. However, the Doppler frequency shift of high-speed moving RFID tags was not analyzed in this paper. Future research efforts may focus on calibrating the Doppler frequency shifts at different vehicle speeds.

## Figures and Tables

**Table 1:** Parameter settings.

Parameter			Value
Robot operation speed			0.3 m/s
Reader read power			30 dbm
Frequency difference			20 MHz
Antenna center/Tag height			110 cm
Antenna’s location	Antennas number	2	(−15,0) (250,265)
		3	(−15,0) (15,0) (250,265)
		4	(−15,0) (15,0) (250,235) (250,265)

**Table 2:** Classification results and weight assignment results.

Category	I	II	III	IV	V	VI	VII
Distance (cm)	15–50	50–150	150–200	200–250	250–300	300–400	400–500
$RMSE_{RSSI}:d_{error}\;(\mathrm{cm})$	5.8057	13.2231	28.8583	100.2913	49.3106	102.8014	136.9578
$RMSE_{Phase}:d_{error}\;(\mathrm{cm})$	10.7603	13.2051	10.6207	11.7970	10.7731	12.8089	13.1522
$\omega_{RSSI}$	0.69954	0.48486	0.18902	0.06525	0.12930	0.05079	0.08762
$\omega_{Phase}$	0.30046	0.51514	0.81098	0.93475	0.87070	0.94921	0.02762

**Table 3:** The average RMSE obtained for each setup.

Antennas Number		2			3			4		
	RF Characteristic	RSSI	Phase	RSSI& Phase	RSSI	Phase	RSSI& Phase	RSSI	Phase	RSSI& Phase
RMSE (cm)										
Route										
Linear		65.1632	26.4752	24.0702	63.3652	27.2659	24.9967	66.1548	18.9315	16.5262
Curve		67.2648	31.2658	28.6988	64.8625	28.1593	21.5334	70.1325	19.1564	15.1935
Turnaround		70.9852	29.3282	26.4143	65.9548	27.5951	19.3431	68.1475	18.2368	15.1972

## Data Availability

No applicable.
